# PHYSICAL ACTIVITY, FALLS, AND FALL INJURIES: THE STUDY OF WOMEN’S HEALTH ACROSS THE NATION

**DOI:** 10.1093/geroni/igz038.1986

**Published:** 2019-11-08

**Authors:** Kelly Ylitalo, Kelley Gabriel

**Affiliations:** 1 Baylor University, Waco, Texas, United States; 2 University of Texas Health Science Center-Houston, School of Public Health-Austin Campus, Austin, Texas, United States

## Abstract

Physical activity (PA) is positively associated with physical functioning, but relations of domain-specific PA with falls is less clear. In 2015, women (n=1,962) self-reported fall history and PA. The Kaiser Physical Activity Survey (KPAS) assessed sports, non-sports leisure, household/caregiving, and total activity. Risk ratios (RR) were generated from poisson regression. One-third (29.7%) of women (mean age=65.1 years [SD 2.7]) reported ≥1 fall and 18.3% reported ≥1 fall injury in the previous 12 months. Women in the highest quartile of sports activity had an increased risk of ≥1 fall (RR=1.19;95%CI:1.00,1.41;p=0.049). Women in the highest quartile of total activity had an increased risk of injurious falls (RR=1.31;95%CI:1.04,1.65;p=0.021), but there was no association for domain-specific indices. KPAS may identify women and activity domains, such as sports, at high risk for falls and injuries. Future work should incorporate device-based PA assessments within domains to understand fall patterns among community-dwelling older adult women.

